# Supplementary material for: Broad-spectrum in vitro antiviral activity of ODBG-P-RVn: an orally-available, lipid-modified monophosphate prodrug of remdesivir parent nucleoside (GS-441524)
Source: bioRxiv. 2021 Aug 10:2021.08.06.455494. Preprint. [Version 2] doi: 10.1101/2021.08.06.455494 (PMC8366795; doi:10.1101/2021.08.06.455494)
Supplement: 1 [file NIHPP2021.08.06.455494V2-supplement-1.pdf]

SUPPLEMENTAL FIGURE LEGENDS

Supplemental Figure S1. Comparison of antiviral activities of RVn, RDV, and ODBG-P-RVn in African green monkey (Vero-E6) cells using reporter-based, image-based, and CPE assays. Representative dose-response inhibition of virus replication by RVn (blue shapes), RDV (black shapes), and ODBG-P-RVn (red shapes). Signal from infected cells treated with DMSO served as 100% fluorescence intensity signal for reporter assays and 100% fluorescence-positive cell counts for image-based assays. CPE inhibition was measured by determining cellular ATP levels using CellTiterGlo 2.0 assay reagent. ATP levels in uninfected cells treated with DMSO served as 100% CPE inhibition. Dose-response curves were fitted to the mean value of experiments performed in biological triplicate for each concentration in the 8-point, 3-fold dilution series using a 4-parameter non-linear logistic regression curve with variable slope. Data points and error bars indicate the mean value and standard deviation of 3 biological replicates; each

bioRxiv preprint doi: https://doi.org/10.1101/2021.08.06.455494; this version posted August 10, 2021. The copyright holder for this preprint (which was not certified by peer review) is the author/funder. This article is a US Government work. It is not subject to copyright under 17 USC 105 and is also made available for use under a CC0 license.

colored shape/line in the legend represents an independent experiment performed in biological triplicate.

Supplemental Figure S2. Comparison of antiviral activities of RVn, RDV, and ODBG-P-RVn in Huh7 cells using reporter-based, image-based, and CPE assays. Representative dose-response inhibition of virus replication by RVn (blue shapes), RDV (black shapes), and ODBG-P-RVn (red shapes). Signal from infected cells treated with DMSO served as 100% fluorescence intensity signal for reporter assays and 100% fluorescence-positive cell counts for image-based assays. CPE inhibition was measured by determining cellular ATP levels using CellTiterGlo 2.0 assay reagent. ATP levels in uninfected cells treated with DMSO served as 100% CPE inhibition. Dose-response curves were fitted to the mean value of experiments performed in biological triplicate for each concentration in the 8-point, 3-fold dilution series using a 4-parameter non-linear logistic regression curve with variable slope. Data points and error bars indicate the mean value and standard deviation of 3 biological replicates; each colored shape/line in the legend represents an independent experiment performed in biological triplicate.

Supplemental Figure S3. Comparison of antiviral activities of RVn, RDV, and ODBG-P-RVn in human bronchioalveolar carcinoma (NCI-H358) cells using reporter-based, image-based, and CPE assays. Representative dose-response inhibition of virus replication by RVn (blue shapes), RDV (black shapes), and ODBG-P-RVn (red shapes). Signal in infected cells treated with DMSO served as 100% fluorescence intensity signal for reporter assays and 100% fluorescence-positive cell counts for image-based assays. CPE inhibition was measured by determining cellular ATP levels using CellTiterGlo 2.0 assay reagent. ATP levels in uninfected cells treated with DMSO served as 100% CPE inhibition. Dose-response curves were fitted to the mean value of experiments performed in biological triplicate for each concentration in the 8-point, 3-fold dilution series using a 4-parameter non-linear logistic regression curve with variable slope. Data points and error bars indicate the mean value and standard deviation of 3 biological

bioRxiv preprint doi: https://doi.org/10.1101/2021.08.06.455494; this version posted August 10, 2021. The copyright holder for this preprint (which was not certified by peer review) is the author/funder. This article is a US Government work. It is not subject to copyright under 17 USC 105 and is also made available for use under a CC0 license.

replicates; each colored shape/line in the legend represents an independent experiment performed in biological triplicate.

Supplemental Figure S4. Comparison of antiviral activities of RVn, RDV, and ODBG-P-RVn in primary-like human small airway epithelial (HSAEC1-KT) cells using reporter-based, image-based, and CPE assays. Representative dose-response inhibition of virus replication by RVn (blue shapes), RDV (black shapes), and ODBG-P-RVn (red shapes). Signal in infected cells treated with DMSO served as 100% fluorescence intensity signal for reporter assays and 100% fluorescence-positive cell counts for image-based assays. CPE inhibition was measured by determining cellular ATP levels using CellTiterGlo 2.0 assay reagent. ATP levels in uninfected cells treated with DMSO served as 100% CPE inhibition. Dose-response curves were fitted to the mean value of experiments performed in biological triplicate for each concentration in the 8-point, 3-fold dilution series using a 4-parameter non-linear logistic regression curve with variable slope. Data points and error bars indicate the mean value and standard deviation of 3 biological replicates; each colored shape/line in the legend represents an independent experiment performed in biological triplicate.

S1.

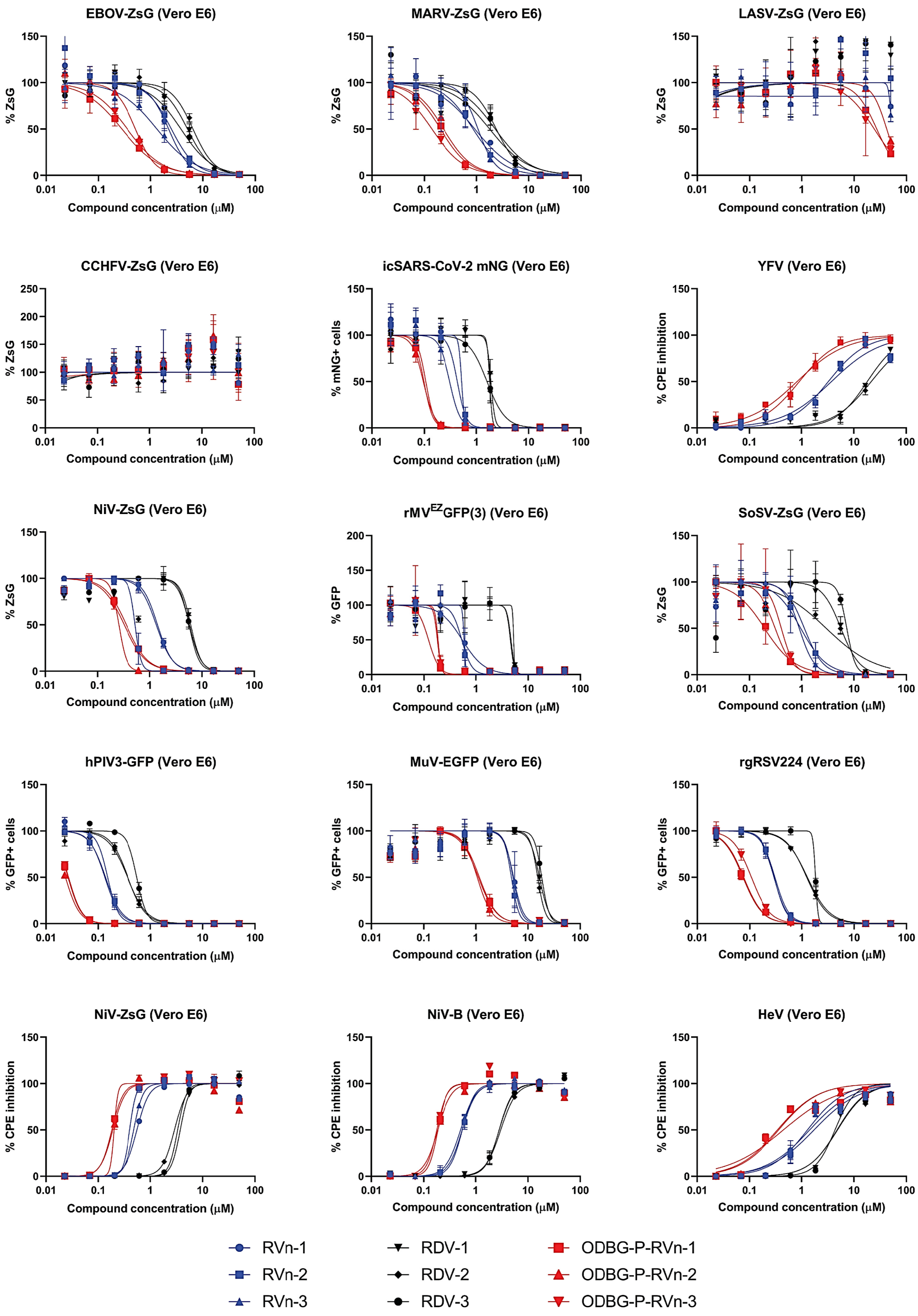

Supplemental Figure S1. Comparison of antiviral activities of RVn, RDV, and ODBG-P-RVn in African green monkey (Vero E6) cells using reporter-based, image-based, and cytopathic effect assays. Representative dose response inhibition of virus replication by RVn (blue shapes), RDV (black shapes), and ODBG-P-RVn (red shapes). Infected cells treated with DMSO served as 100% fluorescence intensity signal for reporter assays and 100% fluorescence-positive cell counts for image-based assays. Inhibition of cytopathic effect was measured by levels of cellular ATP using CellTiterGlo 2.0 assay reagent (Promega, WI). Uninfected cells treated with DMSO served as 100% CPE inhibition. Dose response curves were fitted to the mean value of experiments performed in biological triplicate for each concentration in the 8-point 3-fold dilution series using a 4-parameter non-linear logistic regression curve with variable slope. Data points and error bars indicate the mean value and standard deviation of 3 biological replicates; each colored shape/line in the legend represents an independent experiment performed in biological triplicate.

bioRxiv preprint doi: https://doi.org/10.1101/2021.08.06.455494; this version posted August 10, 2021. The copyright holder for this preprint (which was not certified by peer review) is the author/funder. This article is a US Government work. It is not subject to copyright under 17 USC 105 and is also made available for use under a CC0 license.

S2.

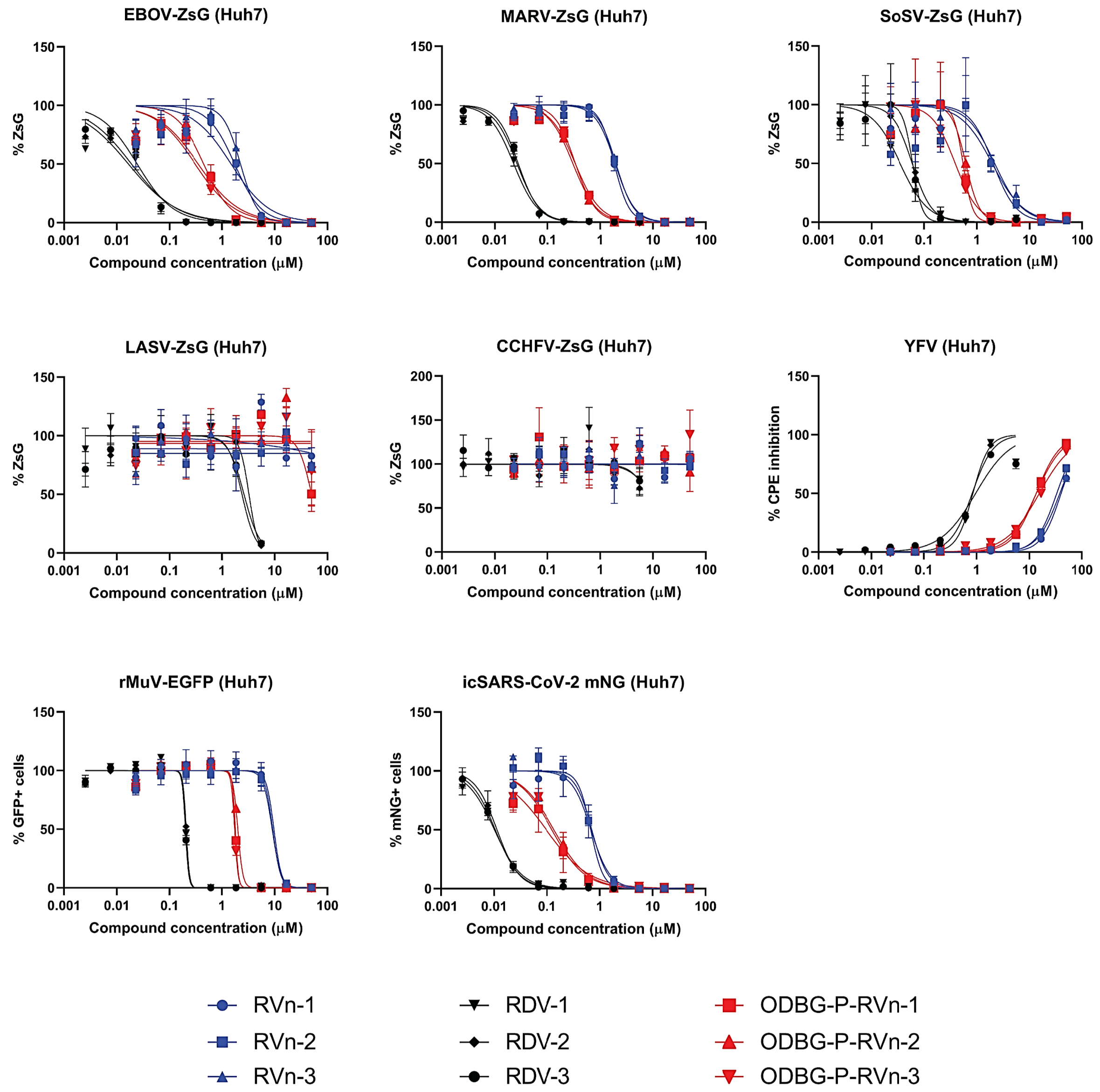

Supplemental Figure S2. Comparison of antiviral activities of RVn, RDV, and ODBG-P-RVn in human hepatoma (Huh7) cells using reporter-based, image-based, and cytopathic effect assays. Representative dose response inhibition of virus replication by RVn (blue shapes), RDV (black shapes), and ODBG-P-RVn (red shapes). Infected cells treated with DMSO served as 100% fluorescence intensity signal for reporter assays and 100% fluorescence-positive cell counts for image-based assays. Inhibition of cytopathic effect was measured by levels of cellular ATP using CellTiterGlo 2.0 assay reagent (Promega, WI). Uninfected cells treated with DMSO served as 100% CPE inhibition. Dose response curves were fitted to the mean value of experiments performed in biological triplicate for each concentration in the 8-point 3-fold dilution series using a 4-parameter non-linear logistic regression curve with variable slope. Data points and error bars indicate the mean value and standard deviation of 3 biological replicates; each colored shape/line in the legend represents an independent experiment performed in biological triplicate.

bioRxiv preprint doi: https://doi.org/10.1101/2021.08.06.455494; this version posted August 10, 2021. The copyright holder for this preprint (which was not certified by peer review) is the author/funder. This article is a US Government work. It is not subject to copyright under 17 USC 105 and is also made available for use under a CC0 license.

S3.

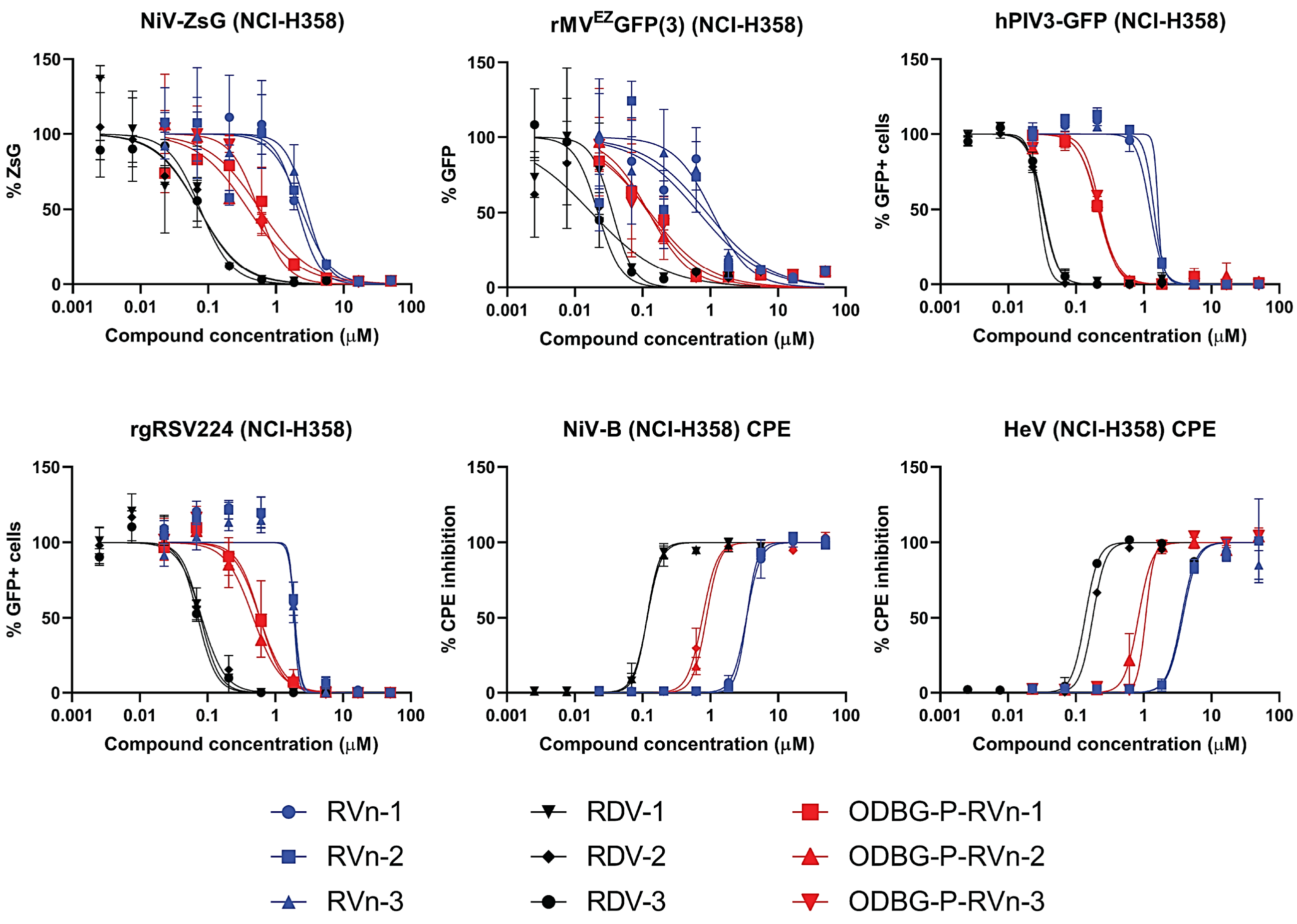

Supplemental Figure S3. Comparison of antiviral activities of RVn, RDV, and ODBG-P-RVn in human bronchioalveolar carcinoma (NCI-H358) cells using reporter-based, image-based, and cytopathic effect assays. Representative dose response inhibition of virus replication by RVn (blue shapes), RDV (black shapes), and ODBG-P-RVn (red shapes). Infected cells treated with DMSO served as 100% fluorescence intensity signal for reporter assays and 100% fluorescence-positive cell counts for image-based assays. Inhibition of cytopathic effect was measured by levels of cellular ATP using CellTiterGlo 2.0 assay reagent (Promega, WI). Uninfected cells treated with DMSO served as 100% CPE inhibition. Dose response curves were fitted to the mean value of experiments performed in biological triplicate for each concentration in the 8-point 3-fold dilution series using a 4-parameter non-linear logistic regression curve with variable slope. Data points and error bars indicate the mean value and standard deviation of 3 biological replicates; each colored shape/line in the legend represents an independent experiment performed in biological triplicate.

bioRxiv preprint doi: https://doi.org/10.1101/2021.08.06.455494; this version posted August 10, 2021. The copyright holder for this preprint (which was not certified by peer review) is the author/funder. This article is a US Government work. It is not subject to copyright under 17 USC 105 and is also made available for use under a CC0 license.

S4.

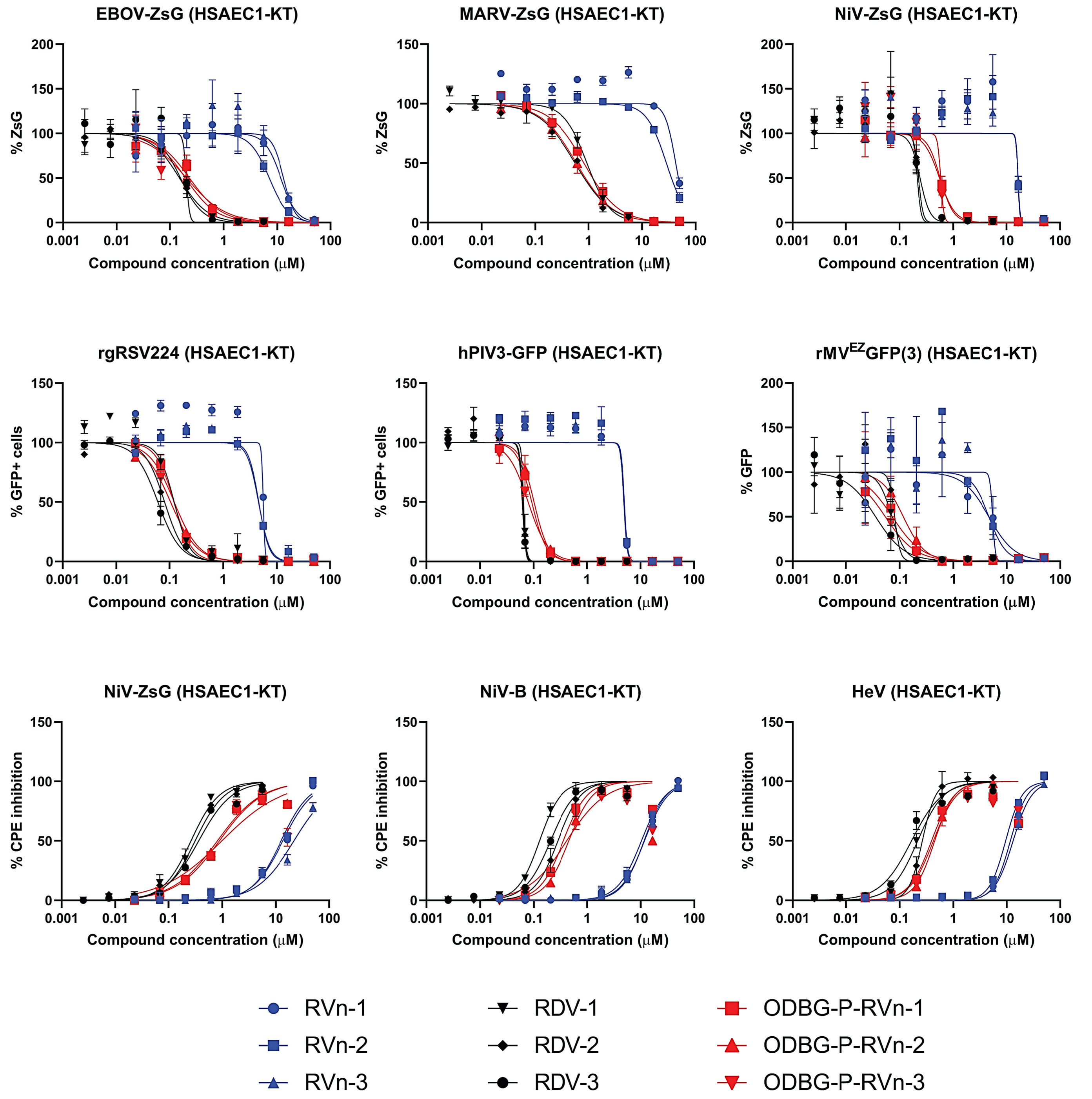

Supplemental Figure S4. Comparison of antiviral activities of RVn, RDV, and ODBG-P-RVn in primary-like human small airway epithelial (HSAEC1-KT) cells using reporter-based, image-based, and cytopathic effect assays. Representative dose response inhibition of virus replication by RVn (blue shapes), RDV (black shapes), and ODBG-P-RVn (red shapes). Infected cells treated with DMSO served as 100% fluorescence intensity signal for reporter assays and 100% fluorescence-positive cell counts for image-based assays. Inhibition of cytopathic effect was measured by levels of cellular ATP using CellTiterGlo 2.0 assay reagent (Promega, WI). Uninfected cells treated with DMSO served as 100% CPE inhibition. Dose response curves were fitted to the mean value of experiments performed in biological triplicate for each concentration in the 8-point 3-fold dilution series using a 4-parameter non-linear logistic regression curve with variable slope. Data points and error bars indicate the mean value and standard deviation of 3 biological replicates; each colored shape/line in the legend represents an independent experiment performed in biological triplicate.

bioRxiv preprint doi: https://doi.org/10.1101/2021.08.06.455494; this version posted August 10, 2021. The copyright holder for this preprint (which was not certified by peer review) is the author/funder. This article is a US Government work. It is not subject to copyright under 17 USC 105 and is also made available for use under a CC0 license.
